# Potential for recovery after extremely prolonged VV-ECMO support in well-selected severe COVID-19 patients: a retrospective cohort study

**DOI:** 10.1186/s12890-023-02836-3

**Published:** 2024-01-08

**Authors:** Jean-Marc de Walque, Christophe de Terwangne, Raphaël Jungers, Sophie Pierard, Christophe Beauloye, Fatima Laarbaui, Melanie Dechamps, Luc Marie Jacquet

**Affiliations:** 1 Department of Cardiovascular Intensive Care, Cliniques Universitaires Saint Luc, Catholic University of Louvain, Avenue Hippocrate 10, Brussels, 1200 Belgium; 2 Emergency Department, Universitair Ziekenhuis Brussels, Brussels, Belgium; 3 Institute for Information and Communication Technologies, Electronics, and Applied Mathematics, Université Catholique de Louvain, Louvain-La-Neuve, Belgium; 4 Pôle de Recherche Cardiovasculaire, Institutde Recherche Expérimentale et Clinique, Université Catholique de Louvain, Brussels, Belgium

**Keywords:** COVID-19, Acute respiratory distress syndrome (ARDS), COVID-19 related ARDS, Extracorporeal membrane oxygenation ( ECMO), Prolonged ECMO

## Abstract

**Background:**

VenoVenous ExtraCorporeal Membrane Oxygenation (VV-ECMO) has been widely used as supportive therapy for severe respiratory failure related to Acute Respiratory Distress Syndrome (ARDS) due to coronavirus 2019 (COVID-19). Only a few data describe the maximum time under VV-ECMO during which pulmonary recovery remains possible. The main objective of this study is to describe the outcomes of prolonged VV-ECMO in patients with COVID-19-related ARDS.

**Methods:**

This retrospective study was conducted at a tertiary ECMO center in Brussels, Belgium, between March 2020 and April 2022. All adult patients with ARDS due to COVID-19 who were managed with ECMO therapy for more than 50 days as a bridge to recovery were included.

**Results:**

Fourteen patients met the inclusion criteria. The mean duration of VV-ECMO was 87 ± 29 days. Ten (71%) patients were discharged alive from the hospital. The 90-day survival was 86%, and the one-year survival was 71%. The evolution of the patients was characterized by very impaired pulmonary compliance that started to improve slowly and progressively on day 53 (± 25) after the start of ECMO. Of note, four patients improved substantially after a second course of steroids.

**Conclusions:**

There is potential for recovery in patients with very severe ARDS due to COVID-19 supported by VV-ECMO for up to 151 days.

## Introduction

Coronavirus disease 2019 (COVID-19) is a viral infection caused by the Severe Acute Respiratory Syndrome Coronavirus 2 virus (SARS-CoV-2). SARS-CoV-2 is an airborne virus that can trigger a variable systemic inflammatory response. Consequently, COVID-19 can manifest with a wide range of symptoms, from asymptomatic infection to critical illness with lung failure due to Acute Respiratory Distress Syndrome (ARDS) [1, 2]. In the most severe cases of lung failure, mechanical ventilation is required, and VenoVenous ExtraCorporeal Membrane Oxygenation (VV-ECMO) support may become necessary [3, 4]. Indeed, growing evidence suggests a potential survival benefit of VV-ECMO in COVID-19-Related ARDS (CARDS) [3, 5–7]. COVID-19 emerged as a pandemic in early 2020, and the high number of patients has led to an increased use of ECMO worldwide [8]. While distinct from influenza- or sepsis-related ARDS due to the specific inflammatory response and coagulopathy associated with COVID-19, CARDS shares similarities in its presentation with a very severe inflammatory response, also known as a “cytokine storm” and a lung histological pattern showing diffuse alveolar damage [9–15]. This may explain why it appeared quite early that ECMO durations for COVID-19 patients were longer than usual.

According to pre-pandemic experience, VV-ECMO has been used in CARDS either as a bridge to recovery or, in some centers, as a bridge to lung transplantation (LT) because lung damage was deemed irreversible or because the benefit of lung transplantation was considered to outweigh the risk of remaining on VV-ECMO. The delays between the start of the disease and the decision to register the patients on the waiting list varied from center to center but were usually between four and eight weeks [16–19]. In a cohort of 12 CARDS patients treated with LT with a median VV-ECMO duration of 49 days, seven were still alive after a follow-up of 32 to 160 days [17]. In another cohort of 11 CARDS patients and one ARDS following bacterial pneumonia, all treated with VV-ECMO (median VV-ECMO duration of 94 days), eight patients could be weaned from VV-ECMO with either a bridge to LT (6 patients) or a bridge to recovery (2 patients). At six months, seven patients were alive [19]. Given the uncertain prognosis of CARDS, the assignment of CARDS patients on VV-ECMO to lung transplantation is highly questionable [20–22].

There is little data on the maximum time delay after which CARDS recovery should no longer be considered possible for VV-ECMO patients. Whereas no study compared the lung healing of patients maintained on ECMO with patients allocated to LT, several retrospective cohorts suggest that lung healing can still occur beyond four to eight weeks on VV-ECMO support. First, in a cohort of ten patients considered “long haulers” on VV-ECMO with a mean ECMO duration of 86 days (range of 42 to 201 days), seven patients were discharged from the intensive care unit (ICU), six of whom recovered and one underwent LT [23]. Second, from a cohort of 12 patients who underwent VV-ECMO for more than 50 days, a 90-day survival rate of 50% has been described [24].

Understanding the possible time delay to recovery delay for CARDS patients plays a crucial role in making informed decisions regarding the continuation of ECMO support and, for eligible candidates, lung transplant allocation.

The present study aims to describe the one-year outcome of patients supported by VV-ECMO for more than 50 days as a bridge to recovery.

## Methods

### Study design

This is a retrospective monocenter study conducted at Saint-Luc University Hospital in Brussels, Belgium. This study was approved on August 12,2020 by the institutional ethics commitee (Comité d’éthique hospital-facultaire des Cliniques Universitaires Saint-Luc: 2020/12AOU/408) and due to the nature of this retrospective study and the preserved anonymity of patients awaiver of informed consent was obtained from the same committee.

### Study cohort

All patients who received VV-ECMO for more than 50 days for CARDS were included between March 2020 and April 2022. A 1-year follow-up was performed.

COVID-19 was diagnosed by a positive result on polymerase chain reaction testing of a nasopharyngeal swab and a typical thoracic computed tomography scanner picture. ARDS was defined according to the Berlin definition [25] with bilateral radiological pulmonary infiltrates and absence of clinical evidence of elevated left atrial pressure.

The indications for VV-ECMO at our institution were initially based on the then-current 2017 Extracorporeal Life Support Organization (ELSO) Guidelines for Adult Respiratory Failure Managed with VV-ECMO [26], then on its revised version for COVID-19 patients [4]. The most common indications were the presence of a respiratory acidosis (arterial pH < 7.25) or a hypoxemic respiratory failure defined as a ratio between partial arterial oxygen pressure and inspired oxygen fraction (PaO_2_/FiO_2_) below 60 despite the use of mechanical ventilation (MV), prone position and inhaled nitric oxide (NO). All patients received lung-protective mechanical ventilation with tidal volumes (Vt) kept below six mL/kg (ideal body weight) and plateau pressure at a maximum of 30 cm H_2_O. Common reasons that precluded using ECMO were a combination of an age limit of 65 years with significant pre-existing comorbidities such as renal, cardiac, or respiratory failure. Although some studies suggest that the time from intubation to cannulation in CARDS should be less than seven days [27], data on this topic are conflicting [28, 29]. Therefore, we did not exclude patients based on these criteria.

All cannulation procedures were percutaneous and initially by femoro-jugular access. The centrifugal pumps were either the Revolution® (Sorin, Italy) or Ecmolife® (Eurosets, Medolla, Italy), and the oxygenators were A.L.ONE ECMO® (Eurosets, Medolla, Italy) or Hilite 7000® (Medos). Blood flow and sweep gas parameters were set according to the ELSO guidelines [26].

### Data collection

Data were collected from the electronic medical records of Saint-Luc University Hospital in Brussels, Belgium. Baseline characteristics included dates of admission to the hospital and to the ICU, age, gender, blood group, medical history, presence of risk factors for COVID-19, presence of coexisting chronic disease, weight, and height to calculate the body mass index (BMI), initial severity assessed by the Sequential Organ Failure Assessment (SOFA) Score [30]. The periods of hospitalization and MV before starting ECMO were also included. Respiratory parameters, namely the PaO_2_/FiO_2_ ratio, Vt, the fraction of delivered O_2_ gas (FDO2), and extracorporeal support operational characteristic, were also collected over time.

### Statistical analysis

Statistical analysis was performed using R (version 4.2.2), R Core Team, Vienna, Austria, 2022. Categorical variables were expressed as counts with percentages and continuous variables as mean and standard deviation or median and 25th–75th percentiles as appropriate. To calculate the mean time from VV-ECMO onset to Vt improvement, the mean values of the shift points of each Vt curve over time were computed using Pettitt’s test for change point detection [31]. The time of weaning from FDO_2_ is when FDO_2_ has been permanently maintained below 100%.

## Results

Out of 313 CARDS patients admitted to the ICU during the study period, 47 (15%) required VV-ECMO support. Of these patients, 33 (70%) spent less than 50 days on ECMO, while the remaining 14 (30%) remained on ECMO for more than 50 days. At the one-year follow-up, the overall mortality rate for the CARDS cohort on VV-ECMO was 60% (28 out of 47 patients)as compared to 29% in the present cohort. The baseline characteristics of the 14 patients included in the study are summarized in Table 1. The mean age was 49 years, with most of the patients (64%) being obese (BMI ≥ 30 kg/m^2^) with a mean BMI of 33 (± 7) kg/m^2^. Four patients (28%) were not known with any comorbidities. Arterial hypertension was the most common comorbidity (36%), followed by diabetes (29%) and hypothyroidism. Immunodepression, ischemic cardiomyopathy, inflammatory bowel disease, chronic bronchitis, pulmonary fibrosis, and sleeve gastrectomy were each present in one patient (7%). One suffered a deep venous thrombosis in the past. The mean SOFA score was 5 (± 2) at admission to the ICU.

**Table 1:** Demographics and characteristics of patients with CARDS for more than 50 days on VV-ECMO

**Gender**	
Female	6 (43%)
Male	8 (57%)
**Age (years)**	49 (±11)
**BMI (kg/m**^**2**^**)**	33 (±7)
**Comorbidities**	
None	4 (28%)
Arterial Hypertension	5 (36%)
Diabetes	4 (29%)
Immunocompromised	1 (7%)
Cardiomyopathy	1 (7%)
Others^a^	6 (43%)
**Blood group**	
A	7 (50%)
B	1 (7%)
O	6 (43%)
AB	0 (0%)
**Treatment before VV-ECMO initiation**	
Dexamethasone	13 (93%)
NO inhalation	10 (71%)
Neuromuscular blocking agent	14 (100%)
Prone position	14 (100%)
**Organ failures**	
Lowest PaO_2_/FiO_2_ ratio before VV-ECMO initiation	51 (±16)
SOFA score	5 (±2)
**Disease course (days)**	
Time from hospital admission to ICU admission	3 (0—7)
Time from hospital admission to MV	6 (2—10)
Time from ICU admission to MV	1 (0—4)
Hospital length-of-stay before VV-ECMO initiation	11 (8—14)
ICU length-of-stay before VV-ECMO initiation	6 (4—8)
Duration of MV before VV-ECMO initiation	5 (5—7)

Results are numbers of patients and percentages, mean values and standard deviation or median and 25th–75th percentiles as appropriate

*Abbreviations*: *BMI* body mass index, *ECMO* Extra Corporeal Life Support, *ICU* Intensive Care Unit, *MV* mechanical ventilation, *NO* nitric oxide, *PaO*_*2*_*/FiO*_*2*_ ratio between arterial pressure and inspired fraction of oxygen, *SOFA* Sequential Organ Failure Assessment

^a^Other comorbidities: bronchitis, deep vein thrombosis, hypothyroidism, inflammatory bowel disease, pulmonary fibrosis, sleeve gastrectomy

Oxygenator, pump, or overall ECMO circuit changes occurred on average five times during the ICU stay. All patients suffered from superinfections, most often bacterial pneumonia, and from recurrent pleural effusions. The main other complications included eight patients (56%) with septic shock, six patients (43%) with acute kidney injury requiring renal replacement therapy, three patients (21%) with pneumothorax, and three patients (21%) with right ventricular failure.

Two patients underwent conversion from venovenous to venoarterial ECMO. The first conversion lasted three days, the indication being a transient left ventricular failure. The second conversion was required after a right ventricular failure complicated by malignant ventricular arrhythmias, prolonged cardiac arrest, and subsequent bilateral ventricular failure, and lasted 64 days. For both patients, heart failure resolved before respiratory failure, and the latter determined the timing of weaning from ECMO.

**Fig. 1:**
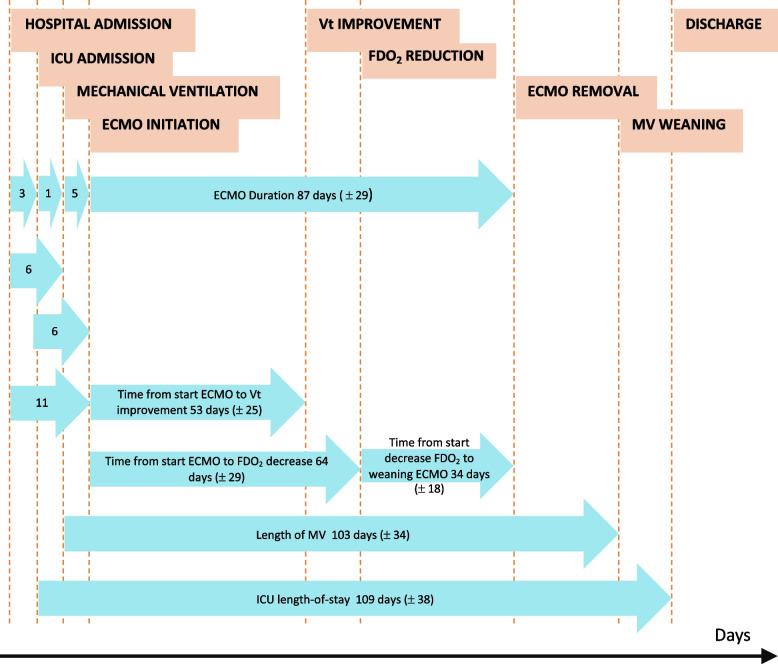
Timeline describing main steps of patients’ hospital stay and their respective durations. Values are means with standard deviations. Abbreviations: ECMO: Extra Corporeal Life Support, FdO2: fraction delivered oxygen (in ECMO sweep gas), ICU: Intensive care unit, MV: Mechanical Ventilation

The median duration of hospitalization before ICU admission was 3 (0—7) days, and patients were intubated 1 (0—4) days after ICU admission. Initiation of VV-ECMO occurred 5 (5—7) days after the institution of MV. The mean VV-ECMO duration was 87 ± 29 days, and patients remained on mechanical ventilation for a mean of 103 ± 34 days. The ICU length of stay was 109 ± 38 days. The main steps of patients' hospital stay and their respective durations can be found in Fig. 1 depicting the temporal evolution of 14 CARDS patients supported by V-V ECMO for over 50 days. The average time from ECMO initiation to VT improvement was 53 days, the average time from ECMO initiation to FDO_2_ reduction was 64 days, and the mean ECMO support duration was 87 days. A total of 11 patients (79%) were weaned from ECMO and discharged from ICU, while 10 (71%) survived until hospital discharge. 90-day survival after ICU admission was 86%, and one-year survival was 71%. Of the four deaths, three occurred in the ICU due to cerebral hemorrhage, septic shock, or right ventricular failure. The fourth death was a sudden cardiac arrest that occurred one week after discharge from the ICU. Patients’ outcomes are described in Table 2.

**Table 2:** Patients outcome during and after VV-ECMO support for more than 50 days

**Disease course**	
Second high dose steroid treatment	4 (29)
ECMO circuits or oxygenator changes	4 (±3)
Length of MV (days)	103 (±34)
Time from start ECMO to Vt improvement (days)	53 (±25)
Time from start ECMO to FDO_2_ decrease (days)	64 (±29)
Time from start decrease FDO_2_ to weaning ECMO (days)	34 (±18)
ECMO duration (days)	87 (±29)
ICU length-of-stay (days)	109 (±38)
**Complications**	
Bacterial pneumonia	14 (100%)
Ischemic stroke	1 (7%)
Cerebral bleeding	1 (7%)
Acalculous cholecystitis	1 (7%)
Renal failure	6 (43%)
Pneumothorax	3 (21%)
Pleural effusions	14 (100%)
Hemothorax	1 (7%)
Septic shock	8 (56%)
Right ventricular failure	3 (21%)
**Outcomes**	
90-day survival	12 (86%)
Weaned from ECMO	11 (79%)
Discharge from hospital	10 (71%)
1-year survival	10 (71%)

Results are numbers of patients and percentages or mean values and standard deviation

*Abbreviations*: *ECMO* Extra Corporeal Life Support, *ICU* Intensive Care Unit, *FDO*_*2*_ fraction delivered oxygen in ECMO sweep gas, *MV* mechanical ventilation, *Vt* tidal volume

**Fig. 2:**
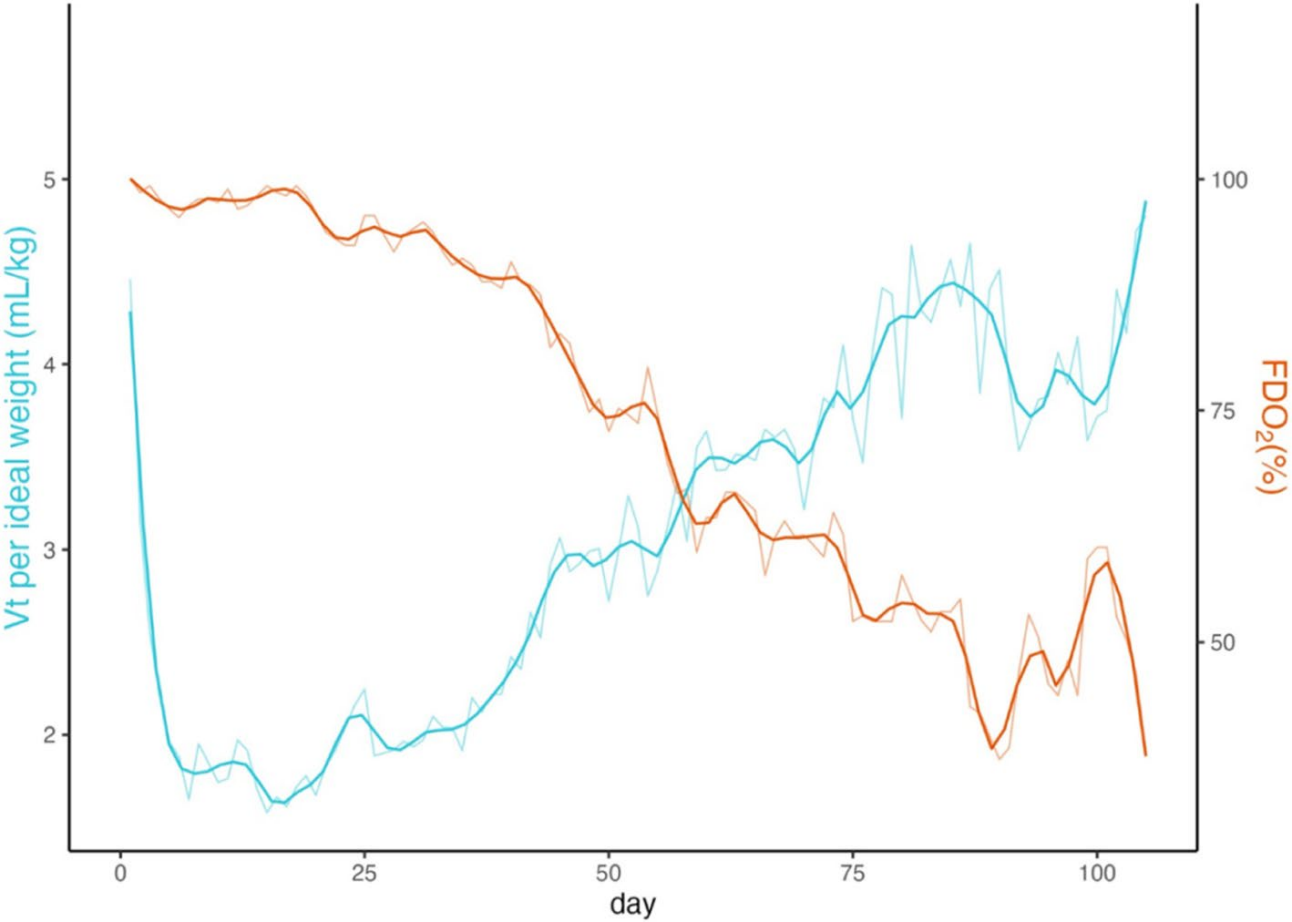
Evolution of tidal Volume (Vt) and Fraction of Delivered Oxygen (FD0_2_) during the VV-ECMO course. Illustrates the significant decrease in Vt promptly after ECMO initiation, followed by a gradual but persistent improvement over weeks, accompanied by a decline in FDO_2_ requirement. Abbreviations: Vt: Tidal Volume, FD0_2_: Fraction of Delivered Oxygen in ECMO sweep gas. The blue curves represent the mean Vt over time (left y-axis), while the red curve represents the mean FDO2 over time (right y-axis)

Disease courses on VV-ECMO were characterized by severely impaired lung compliance. With the standardized ventilator setting, mean Vt per kilogram (kg) was 4.46 mL/kg at VV-ECMO initiation, and 1.6 mL/kg, 2.4 mL/kg, 3.4 mL/kg, 3.7 mL/kg, and 4.8 mL/kg at 20, 40, 60, 80 and 105 days respectively. Figure 2 shows the evolution of Vt/kg of ideal weight during the ICU stay. The start of improvement of Vt occurred 53 ± 25 days after VV-ECMO onset and the beginning of the decrease of the FDO_2_ 11 days later, 64 ± 29 days after VV-ECMO onset.

**Fig. 3:**
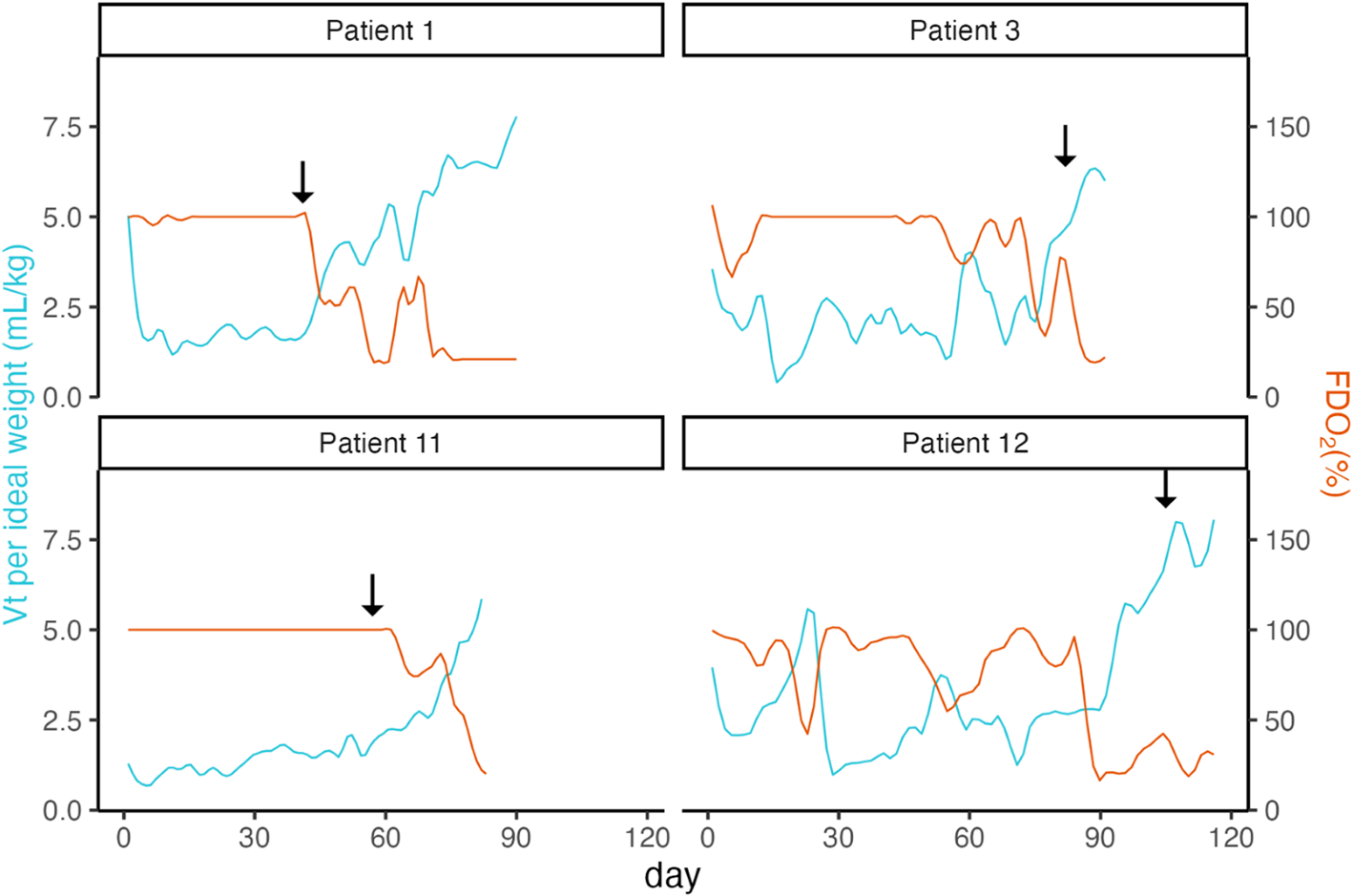
Tidal volume (Vt) evolution in four patients treated with a second course of steroids. The blue curves represent the mean Vt over time (left y-axis), while the red curve represents the mean FDO2 over time (right y-axis). Arrows represent the start of the second course of steroids. Abbreviations: Vt: Tidal Volume. FD0_2_: Fraction of Delivered Oxygen in ECMO sweep gas

Of the entire cohort, 13 patients (93%) were treated with an initial 10-day course of Dexamethasone. Four subsequently received a second course of steroids, as their condition did not improve despite the absence of complications, especially infections. Infections were screened by daily blood cultures, bronchoalveolar lavage, and plasma PCRs for at least cytomegalovirus and herpes detection. Treatment consisted of methylprednisolone 1–2 mg/kg daily for three days, followed by a tapering regimen at the physician's discretion. The second course of corticoid treatment was initiated at 6, 8, 12, and 15 weeks after initiation of VV-ECMO. In all these patients, treatment was associated with improved oxygen requirements (decreasing FDO2) and increased Vt within one week. ECMO could be weaned within two to seven weeks (Fig. 3).

## Discussion

For well-selected patients, this study describes a considerable potential for recovery for severe CARDS cases supported by VV-ECMO for more than 50 days.

The in-hospital mortality of 29% is low compared with other study cohort [32]. The low mortality rate is certainly influenced by the strict selection of patients aged under 65 years without pre-existing organ failure or significant comorbidity, meeting the criteria described for VV-ECMO consideration for CARDS-related respiratory failure [4]. Moreover, a selection bias occurred by excluding patients deceased before day 50 on ECMO. Nevertheless, these results suggest that patients with CARDS can be supported with prolonged VV-ECMO and that lung recovery might still occur beyond 4 to 8 weeks.

The findings of this study call into question the indication for LT for CARD patients. Consistent with the present findings, a cohort of 16 patients listed for LT but never transplanted showed a recovery rate (namely hospital discharge) of 56% (nine patients) while on the waiting list. Median ECMO duration was 94 (58.5–126.5) days. The cohort shared similar baseline characteristics to the present one, with a selection bias of patients allocated to LT after a median of 67 (38.0–112.25) days on ECMO, a median age of 49.5 years, and a median BMI of 30.5. Younger age (44.0 versus 61.0 years) was associated with recovery, compared with patients who died [33]. Furthermore, although LT reduces the risk of ECMO-related complications, it is accompanied by its own morbidity and mortality. Consequently, given the scarcity of organs suitable for transplantation, the benefit of LT should be formally demonstrated before going in this direction.

Beyond the time delay required for lung recovery, there is currently no reliable tool predicting CARDS prognosis, making the decision regarding ECMO withdrawal or LT allocation particularly challenging. In a retrospective study, the RESP score [34] used for non-COVID-19 ARDS patients has been shown not to predict survival for CARDS patients [35]. Additionally, determining whether or not pulmonary recovery will be impossible, as is the case for irreversible pulmonary fibrosis, is currently not possible. Here, all patients had a clinical course marked by very impaired lung compliance with a Vt per kilo of ideal weight as low as 1.6 mL/kg on average at day 20 after VV-ECMO initiation All the patients on VV-ECMO were ventilated in a pressure control mode with a plateau pressure kept at maximum 30 cmH_2_O and positive end-expiratory pressure (PEEP) adjusted based on patients individual compliance and recruitablity [36, 37]. In other words, the Vt reported were the highest Vt achievable using the maximum allowable plateau pressure for protective ventilation, which was possible under VV-ECMO assistance [38]. This cohort provides insights that severely impaired lung compliance does not necessarily indicate irreversible lung damage. A comparative prospective and randomized study between prolonged VV-ECMO support and early lung transplant seems not feasible for limited resources and ethical reasons. Instead, prognostic factors assessing the potential for recovery should be established scientifically.

The encouraging possibility of successful recovery after remarkably prolonged ECMO support in CARDS patients challenges existing data and recommendations for non-COVID-19 ARDS patients. A 2019 retrospective study from the ELSO registry defined prolonged ECMO as ECMO support exceeding 14 days. This study reported a survival rate of 51%, with factors associated with survival including younger age, white race, increased body weight, viral/bacterial pneumonia, higher PEEP, NMB, VV-ECMO mode, and decreased time from intubation to ECMO initiation [39]. More recently, a retrospective analysis of 55 patients undergoing ECMO for non-COVID-19 ARDS or during bridging to lung transplantation described 18 patients receiving ECMO support for more than 28 days. Hospital survival showed no significant difference between the short-term and long-term ECMO groups (54% and 50%, respectively). The longest ECMO run time in a survivor was 65 days [40]. In both studies, the duration of VV-ECMO support alone did not serve as a prognostic factor.

Four patients received a second empirical course of steroids, and all had a subsequent improvement, suggesting underlying corticosteroid-sensitive disease. Treatment was initiated when the absence of improvement had no overt etiology and after excluding any infectious cause. It was therefore suspected that the persistent respiratory insufficiency might be linked to an ARDS that was no longer related to COVID-19, and that the pathology could respond to steroids [41, 42]. In line with this, the development of organizing pneumonia after COVID-19 infection has been described in several case-reports [43]. These are only four observations but they point to the need for a more detailed evaluation of the evolution of etiology and underlying histology in persistent ARDS.

This study has limitations, including its observational retrospective design and relatively small sample size. Additionally, a potential selection bias has occurred by excluding patients who died before day 50 on ECMO. However, the study's strength lies in the highly standardized management of patients at an experienced ECMO center, which facilitated a detailed analysis of Vt and FDO_2_ trends over time.

## Conclusion

For well-selected patients, there is considerable potential for pulmonary recovery in CARDS supported by VV-ECMO for up to 151 days. This raises the question of how long VV-ECMO support should be continued in CARDS, but also in non-COVID-19 ARDS. Although the sample size of this study is limited, it prompts us to reassess lung transplantation criteria and identify prognostic factors that accurately predict the probability of recovery from ARDS on ECMO.

## Data Availability

The datasets used and analyzed during the current study are available from the corresponding author upon reasonable request.

## Abbreviations

AKI: Acute Kidney Injury
AHT: Arterial Hypertension
ARDS: Acute Respiratory Distress Syndrome
BMI: Body Mass Index
COVID-19: Coronavirus Disease 2019
CARDS: Coronavirus Disease 2019 Related Acute Respiratory Distress Syndrome
ECMO: Extracorporeal Membrane Oxygenation
ECLS: Extra Corporeal Life Support
ELSO: Extracorporeal Life Support Organization
FDO_2_: Fraction Delivered Oxygen
ICU: Intensive Care Unit
IQR: InterQuartile Range
Kg: Kilogram
LOS: Length Of Stay
LT: Lung Transplantation
MV: Mechanical Ventilation
NO: Nitric Oxide
PaO_2_/FiO_2_: Ratio between arterial pressure and the inspired fraction of oxygen
PEEP: Positive End-Expiratory Pressure
SARS-CoV-2: Severe Acute Respiratory Syndrome Coronavirus 2
SOFA: Sequential Organ Failure Assessment
Vt: Tidal volume
VV-ECMO: Venovenous Extracorporeal Membrane Oxygenation
